# Infantile Extracranial Rhabdoid Tumor of the Scalp

**DOI:** 10.1155/2021/6682960

**Published:** 2021-05-11

**Authors:** Sura Al Rawabdeh, Deifallah Alsharari, Hayat Khasawneh, Ola M. Al Waqfi, Qamar Yaser Malabeh, Hiathem Abu Alhaija, Raed Mohammad Aljubour, Hamzeh M. Alkhawaldeh

**Affiliations:** ^1^Department of Pathology, Princess Iman Center for Research and Laboratory Sciences, King Hussein Medical Center, Amman, Jordan; ^2^Royal Rehabilitation Center, King Hussein Medical Center, Amman, Jordan; ^3^Department of Radiology, King Hussein Medical Center, Amman, Jordan; ^4^Department of Dermatology, King Hussein Medical Center, Amman, Jordan; ^5^Department of Neurosurgery, King Hussein Medical Center, Amman, Jordan

## Abstract

Extracranial rhabdoid tumor is a rare tumor that can originate in multiple organs, and it is most commonly seen in the kidneys. This tumor has a grave prognosis. We report to the best of our knowledge the first case of infantile scalp extracranial rhabdoid tumor in a 6-month-old male baby who presented with a right parietal scalp mass since the age of 1 month. This mass was initially diagnosed as scalp hemangioma based on clinical and imaging findings. However, this mass was growing fast which necessitated excision. Pathologic examination after excision showed a malignant tumor composed of sheets of rhabdoid cells. Immunohistochemically, this tumor tested positive for vimentin, CD 99, glypican-3, synaptopysin, WT-1, CK, and EMA. INI-1 immunostain was lost in the tumor cells. Subsequently, this tumor was pathologically diagnosed as extracranial scalp rhabdoid tumor. After tumor excision, the patient was referred to pediatric oncology to receive chemotherapy. Experience with scalp extracranial rhabdoid tumors is limited. However, this tumor in other organs carries a grave prognosis. Although scalp extracranial rhabdoid tumor is an extremely rare tumor, it should be kept in mind in the differential diagnosis of infantile scalp masses given the need of combined surgical and chemotherapeutic treatment.

## 1. Introduction

Masses in the head have a broad differential diagnosis and are usually divided into congenital versus acquired. Usually, CT and MRI are used in the evaluation of these masses initially. Congenital causes include, for example, encephaloceles, nasal gliomas, dermoid and epidermoid cysts, and benign tumors, while acquired causes include sarcoma, Langerhans cell histiocytosis (LCH), metastatic neuroblastoma, and infectious or traumatic lesions [1].

Extracranial rhabdoid tumor is a rare malignancy occurring in the pediatric population with a grave prognosis [2]. This tumor extracranially most commonly involves the kidney among other organs including neck, back, retroperitoneum, pelvis, and chest [2, 3].

## 2. Case Presentation

**Figure 1:**
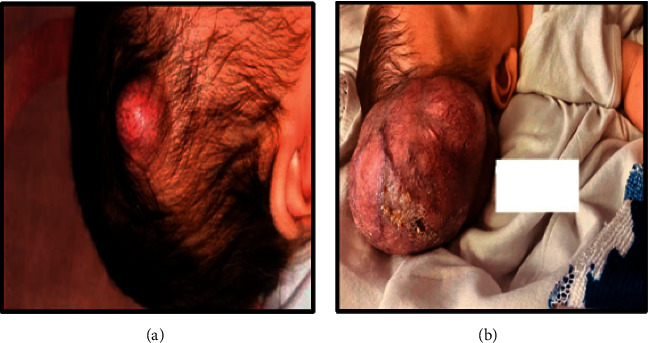
Image of lesion at 1 month (a) and image of lesion at 6 months (b). The mass showed progressive enlargement in size with surface ulcerations.

We present a male premature baby with a gestational age of 33 weeks and part of twin pregnancy who presented with a right scalp parietal lump since the age of 1 month that progressively increased in size over time as shown in Figures 1(a) and 1(b).

**Figure 2:**
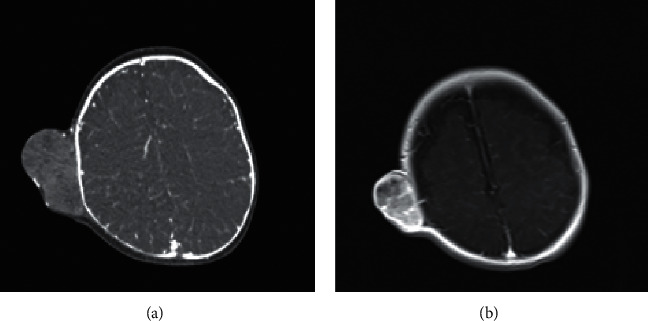
Contrast-enhanced CT demonstrating right parietal vividly enhancing scalp lesion without involvement of the skull (a). Contrast-enhanced MRI also showing vivid enhancement (b).

This lesion was evaluated by contrast-enhanced CT and MRI which showed vivid enhancement as shown in Figures 2(a) and 2(b).

Initial clinical impression was the diagnosis of scalp hemangioma, but given rapid progressive growth, excision of lesion was performed and the specimen was sent to pathology for definitive diagnosis.

**Figure 3:**
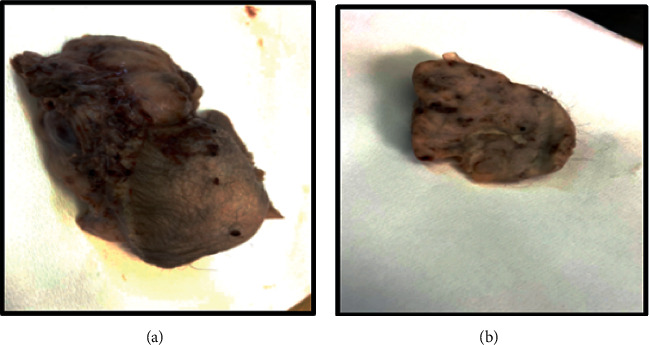
The gross findings showing a skin-covered polypoid firm mass (a) with solid tan-white cut surface (b).

The gross findings were a skin-covered irregular polypoid firm mass measuring 11 × 10 × 9 cm. On sectioning, the cut surface showed solid tan-white appearance as shown in Figures 3(a) and 3(b).

**Figure 4:**
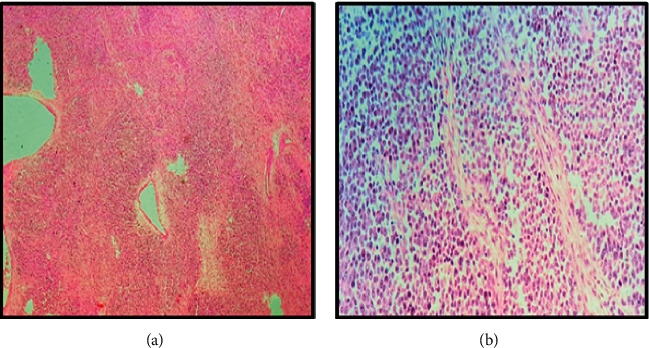
H&E microscopic findings. (a) A low-power view of sheets of polygonal cells with variably fibromyxoid stroma. (b) A high-power view showing the classic rhabdoid profile, comprising abundant cytoplasm with eosinophilic hyaline globules and vesicular nuclei with prominent nucleoli.

Pathologic microscopic evaluation showed skin tissue infiltrated by malignant tumor cells composed of sheets of rhabdoid cells having abundant cytoplasm with eosinophilic hyaline globules, vesicular nuclei, and prominent nucleoli. The background stroma is fibromyxoid with high mitotic activity in addition to areas of necrosis as shown in Figures 4(a) and 4(b).

**Figure 5:**
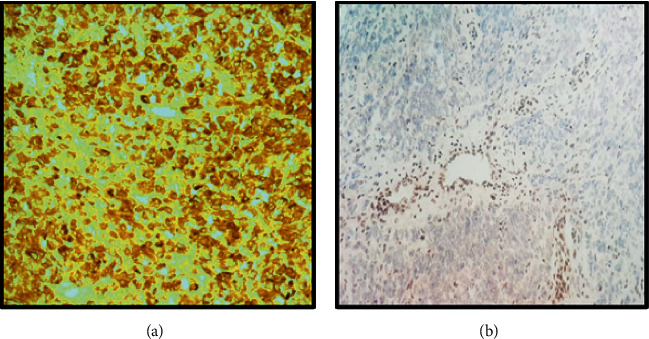
Immunohistochemical findings. (a) EMA showing strong positivity in the tumor cells. (b) Loss of nuclear INI-1 staining in the tumor cells with positive internal control (endothelial cells).

Immunohistochemical stains were performed, and the tumor cells were strongly immunoreactive for vimentin, CD99, glypican-3, synaptopysin, WT-1, CK, and EMA as shown in Figure 5(a).

INI-1 immunostain was lost in the tumor cells as shown in Figure 5(b).

Immunostains for CD31, CD30, desmin, myogenin, factor VIII, SALL-4, Oct ¾, S100, PLAP, ATP, chromogranin, and CD56 were all negative.

The surgical margins were negative, and no second surgical intervention was performed.

Based on microscopic examination and the immunohistochemical profile, a diagnosis of extracranial malignant rhabdoid tumor was made. The patient was referred to pediatric oncology for staging and treatment.

This patient was treated according to the European rhabdoid protocol. However, the patient received just intensive chemotherapy as there was no role for radiotherapy or for autologous bone marrow transplant because of the age of the patient. The intensive chemotherapy given was composed of multiple chemotherapy regimens including the following chemotherapeutic agents: doxorubicin, ifosfamide, carboplatinum, etopside, vincristine, cyclophosphamide and actinomycin-D.

## 3. Discussion

Rhabdoid tumors can involve the CNS primarily and are termed atypical teratoid rhabdoid tumor (ATRT). However, rhabdoid tumors can be extracranial either involving the kidney primarily or involving the soft tissues [4].

Malignant extracranial rhabdoid tumor diagnosis depends on morphologic pathologic features which are nonspecific for this tumor, since the differential diagnosis also includes other tumors such as Ewing's sarcoma, Wilms' tumor, desmoplastic small round cell tumor, clear cell sarcoma, congenital mesoblastic nephroma, synovial sarcoma, undifferentiated sarcoma, rhabdomyosarcoma, and epithelioid sarcoma [5]. Histologically, soft tissue rhabdoid tumors (STRT) show noncohesive single cells, clusters, or sheets of large tumor cells with abundant glassy eosinophilic cytoplasm, an eccentric vesicular nucleus, and an extremely large nucleolus with immunohistochemistry showing positivity for vimentin and/or cytokeratin, and the diagnosis is confirmed with loss of INI-1 stain on immunohistochemical staining [5–7].

Malignant Extracranial rhabdoid tumor has been reported in multiple soft tissue organs such as neck, back, retroperitoneum, pelvis, and chest [2, 3]. We present to the best of our knowledge the first infantile extracranial rhabdoid tumor arising in the scalp although there has been a prior case report of congenital malignant rhabdoid tumor of the scalp by Cobb et al. [4]. After surgical excision and pathologic diagnosis, the patient was referred to pediatric oncology for treatment. Given the rarity of malignant extracranial rhabdoid tumor, standardized treatment protocols have not yet been popularized worldwide. Generally, treatment protocols are based on a multimodal approach, combining surgery, chemotherapy, and radiotherapy. Despite many attempts to improve these various regimens, malignant extracranial rhabdoid tumor is still described as lethal [2, 4, 8]. At our institution, we used the European rhabdoid protocol for treatment, and this particular patient received just intensive chemotherapy given the young patient's age as there was no role for radiotherapy or autologous bone marrow transplant.

## 4. Conclusion

Extracranial rhabdoid tumor is a rare tumor with grave prognosis, and diagnosis is based on histological and immunohistochemistry findings specifically a negative INI-1 immunohistochemical stain. To the best of our knowledge, we report the first infantile extracranial rhabdoid tumor of the scalp even though there has been a prior report of a congenital extracranial rhabdoid tumor of the scalp.

## Data Availability

The data used to support the findings of this study are available from the corresponding author upon request.

## References

[1] Morón F. E., Morriss M. C., Jones J. J., Hunter J. V. (2004). Lumps and bumps on the head in children: use of CT and MR imaging in solving the clinical diagnostic dilemma. *Radiographics*.

[2] Madigan C. E., Armenian S. H., Malogolowkin M. H., Mascarenhas L. (2007). Extracranial malignant rhabdoid tumors in childhood. *Cancer*.

[3] Hong C. R., Kang H. J., Ju H. Y. (2015). Extra-cranial malignant rhabdoid tumor in children: a single institute experience. *Cancer Research and Treatment*.

[4] Cobb A. R. M., Sebire N. J., Anderson J., Dunaway D. (2012). Congenital malignant rhabdoid tumor of the scalp. *Journal of Cranio-Maxillofacial Surgery*.

[5] Hoot A. C., Russo P., Judkins A. R., Perlman E. J., Biegel J. A. (2004). Immunohistochemical analysis of hSNF5/INI1 distinguishes renal and extra-renal malignant rhabdoid tumors from other pediatric soft tissue tumors. *American Journal of Surgical Pathology*.

[6] Sigauke E., Rakheja D., Maddox D. L. (2006). Absence of expression of SMARCB1/INI1 in malignant rhabdoid tumors of the central nervous system, kidneys and soft tissue: an immunohistochemical study with implications for diagnosis. *Modern Pathology*.

[7] Fanburg-Smith J. C., Hengge M., Hengge U. R., Smith J. S. C., Miettinen M. (1998). Extrarenal rhabdoid tumors of soft tissue: a clinicopathologic and immunohistochemical study of 18 cases. *Annals of Diagnostic Pathology*.

[8] Cheng H., Yang S., Cai S. (2019). Clinical and prognostic characteristics of 53 cases of extracranial malignant rhabdoid tumor in children. A single-institute experience from 2007 to 2017. *The Oncologist*.

